# Evaluation of High Resolution Melting analysis as an alternate tool to screen for risk alleles associated with small kidneys in Indian newborns

**DOI:** 10.1186/1471-2369-12-60

**Published:** 2011-10-28

**Authors:** Ashwini Raghavendra, Annes Siji, TS Sridhar, Kishore Phadke, Anil Vasudevan

**Affiliations:** 1 Division of Molecular Medicine, St. John's Research Institute, Sarjapur Road, Bangalore 560034, Karnataka, India; 2 Children's Kidney Care Center, Department of Pediatrics, St. John's Medical College Hospital, Sarjapur Road, Bangalore 560034, Karnataka, India

## Abstract

**Background:**

Single nucleotide polymorphisms (SNPs) are the most common forms of sequence variations in the human genome. They contribute to the human phenotypic spectrum and are associated with variations in response to pathogens, drugs and vaccines. Recently, SNPs in three human genes involved in kidney development (*RET*, *PAX2 *and *ALDH1A2*) have been reported to be associated with variation in renal size and function. These known SNPs could potentially be used in the clinic as markers for identifying babies who may have smaller kidneys and permit close follow up for early detection of hypertension and acquired renal dysfunction. The aim of this study was to evaluate the use of High Resolution Melting technique (HRM) as a tool for detecting the known SNPs in these three genes in comparison to sequencing which is the gold standard.

**Methods:**

High resolution melting analysis was performed on 75 DNA samples that were previously sequenced for the known polymorphisms in *RET *(rs1800860), *PAX2 *(rs11190688) and *ALDH1A2 *(rs7169289) genes. The SNPs were G > A transitions in *RET *and *PAX2 *and A > G in *ALDH1A2 *gene. A blinded assessment was performed on these samples for evaluation of the HRM technique as compared to sequencing.

**Results:**

Each variant had a unique melt curve profile that was reproducible. The shift in melting temperature (Tm) allowed visual discrimination between the homozygous alleles (major and minor) in all three genes. The shape of the melting curve as compared to the major allele homozygous curve allowed the identification of the heterozygotes in each of the three SNPs. For validation, HRM was performed on 25 samples for each of the three SNPs. The results were compared with the sequencing results and 100% correct identification of the samples was obtained for *RET*, *PAX2*, and *ALDA1H2 *gene.

**Conclusion:**

High Resolution Melting analysis is a simple, rapid and cost effective technique that could be used in a large population to identify babies with the risk alleles. These high risk children could be followed up for early detection of hypertension and acquired renal dysfunction.

## Background

Single nucleotide polymorphisms (SNPs) are the most common form of sequence variation in the human genome. Large scale studies have shown that these single base variations in DNA sequences affect the development of some diseases and also provide the genetic bases for individual variations in response to pathogens, drugs and vaccines [1,2]. Therefore, the identification of SNPs may be seen as a component of individualized risk assessment.

Recently, common variants of three human genes involved in kidney development- *RET *(rs1800860), *PAX2 *(rs11190688) and *ALDH1A2 *(rs7169289) have been reported to be associated with variation in renal volume (surrogate marker for nephron numbers) and function, in a cohort of Montreal newborns [3-5]. Understanding the role of these SNPs in renal development will help unravel one of the determinants of wide variation in nephron numbers observed in humans (0.3-1.3 million nephrons per kidney). Brenner et. al. in the late eighties proposed that individuals at the lower end of the nephron endowment spectrum are predisposed to "essential" hypertension and renal insufficiency in later life [6]. This has been supported by observations in autopsy studies in German adults and in Australian cohort of healthy adults [7,8]. The results of these studies may have important clinical implications for the Indian population because, the observed mean kidney volume among babies from Bangalore was found to be only 65% compared to newborns from Montreal, after adjusting for the difference in body size [9]. It is also well known that 25-40% of adult Indians suffer from essential hypertension [10]. These polymorphisms can potentially be used in the clinic as markers for identifying babies who may have small kidneys. This would help in ensuring close follow up for early detection of hypertension and acquired renal dysfunction during late childhood and early adulthood.

One of the challenges in the detection of SNPs in clinical samples is to have methods to identify the SNPs rapidly, accurately, and cost effectively. Many assays have been developed to detect SNPs such as direct sequencing, single-stranded conformation polymorphism analysis, denaturing gradient gel electrophoresis, temperature gradient capillary electrophoresis, restriction enzyme digestion of polymerase chain reaction (PCR) products and sequencing [11-13]. Each of these methods has a different trade off between complexity, sensitivity, turn-around time and cost. High Resolution Melting analysis is a well established, closed tube, rapid and high throughput technique. The analysis depends on DNA melting and annealing in the presence of saturating DNA binding dye. The presence of a single base change in the amplicon influences the thermodynamic stability of the duplex resulting in a slight change in the melting temperature (Tm) and the shape of the melt curve [14,15]. The aim of this study was to evaluate the use of HRM as a tool for detecting known SNPs in the three human genes, *RET*, *PAX2 *and *ALDH1A2 *which affect kidney size in children.

## Methods

### Samples

Cord blood samples stored as part of an ongoing study for understanding the determinants of newborn kidney size were obtained from the division of molecular medicine laboratory at St. John's Research Institute. Ethical approval was obtained prior to commencement of the study. Buffy coat was collected after centrifugation (Centrifuge 5810R, Eppendorf, Hamburg, Germany) at 5000 g for 20 min. DNA was extracted using Promega DNA Wizard Kit. The quantity and quality of DNA were estimated using NanoDrop ND-1000 (Thermo Scientific, India). The samples were previously sequenced (ABI 3730XL) for the known polymorphic variants in *RET *(rs1800860 G > A), *PAX2 *(rs11190688 (G > A), and *ALDH1A2 *(rs7169289, A > G) genes. For validating the assay, twenty five DNA samples for each SNP were randomly selected and a blinded assessment was performed.

### HRM Assay Conditions

Primers were designed with melting temperature (Tm) between 60°C to 65°C using Primer3 plus software to amplify a small fragment (95 - 124 base pairs) encompassing the known polymorphic variants of *RET*, *PAX2 *and *ALDH1A2 *genes (Table 1). In the *RET *gene, there is a second SNP rs9282835 that is positioned 37bp upstream to the rs1800860 SNP. The forward primer was designed such that it does not overlay the rs9282835 in order to avoid inaccurate genotyping of the rs1800860. HPLC purified primer sequences were obtained from Eurofins Genomics India Pvt Ltd (Bangalore, India). The PCR and HRM were performed (in triplicate) in a single run on a LightCycler 480 (Roche Diagnostics, Penzberg, Germany). The reactions were carried out in 96-well plates using either 10ng (*PAX2*) or 20ng of template DNA (*RET *and *ALDH1A2*), 2.5 mM MgCl_2 _(*RET*), 3.0 mM MgCl_2 _(*ALDH1A2 *and *PAX*), 200 nM of each primer in 1X HRM master mix containing ResoLight dye (Roche diagnostics) with PCR grade water adjusted to a total volume of 10 μl. The reaction conditions were as follows: activation step at 95°C for 10 min followed by 45 cycles of 95°C for 10s, 65°C for 15s, and 72°C for 15s. These reaction conditions were empirically determined by optimizing the annealing temperature, MgCl_2 _concentration, quantity of template and number of cycles.

**Table 1:** Primer sequences and amplicon size for *RET, PAX *and *ALDH1A2 *gene with SNP position.

Gene	Forward/Reverse primer	Product size (bp)	SNP position in the amplicon (from 5'end)
*RET*	ATCGGGAAAGTCTGTGTGGACCTAGCGTGCTGCAGTTGGCA	95	33

*PAX2*	GCCCCTTTGTCAACTTTGAGAGCCC TGCCAACCTTGAATTCCTCAAGCA	122	70

*ALDH1A2*	TGGGTGGAGAGCAAAAGAGAGCA GTAGCCTCCCCCGTGCCTGA	124	57

### Melt curve acquisition and analysis

For melt curve acquisition, the PCR products were first heated to 95°C for 1 min and then rapidly cooled to 40°C at 2.2°C per second to allow heteroduplex formation. The PCR products were then reheated to 95°C at 0.01°C per second. Data was acquired between 65°C to 95°C at 50 acquisitions per °C. HRM curve analysis was performed using the LightCycler 480 Software version 1.5 at default settings. The melt curve data for the amplicons were normalized between 0% and 100% fluorescent intensity and temperature shifted (to eliminate temperature difference between samples) for comparisons. Difference plots were generated by converting the normalized and temperature shifted melting profile of the reference sample (major allele sample) to a horizontal line and subtracting the melting profiles of the other samples against the reference sample. Significant differences in fluorescence from the horizontal baseline indicate variations in the dissociation pattern of the amplicons. Differences were judged as significant if the technical triplicate fell outside the range. The genotypes were discriminated visually from normalized melting curves and difference plots. The derivative plot allowed direct visualization of the melting temperatures (Tm) and was used to differentiate between major allele homozygous genotypes and minor allele homozygous genotypes.

## Results

### Standardization of the assay

**Figure 1:**
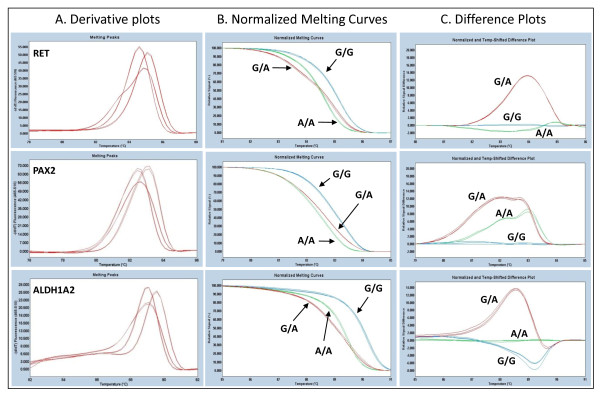
**Standardization assay showing derivative plots (Panel A), normalized melt curves (Panel B) and difference plots (Panel C) for *RET*, *PAX2 *and *ALDH1A2 *genes**. Graph shows three samples for each genotype performed on reference samples.

PCR and HRM were first performed on previously sequenced samples in order to determine the Tm and characterize the melting curve profiles of different genotypes for all three genes. The major allele homozygotes were G/G for *RET *and *PAX2 *and A/A for *ALDH1A2 *while the minor allele homozygotes were A/A for *RET *and *PAX2 *and G/G for *ALDH1A2*. The derivative plots, normalized melt curves and difference plots of samples used for standardization are presented in Figure 1. All the genotypes produced unique melt curves and could be visually discriminated. The larger the difference in the melting temperature, the better is the discrimination between the curves as observed in the normalized melting curves and difference plots.

Heterozygotes could not be distinguished based on the Tm but only by the shape of the melt curve. Heterozygotes form heteroduplexes since re-association occurs randomly after denaturation.The heterozygous samples showed less discrimination to the A/A homozygote than compared to G/G homozygote in the normalized curves. The melt profiles of each of the heterozygous variants in *RET*, *PAX2 *and *ALDH1A2 *genes were clearly distinguishable from the major allele homozygote by the amplitude and the shape of the curves on the difference plots.

### Validation of the assay

**Figure 2:**
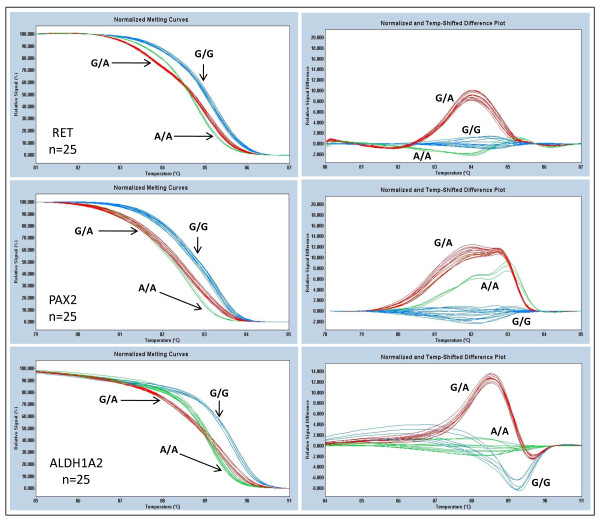
**Validation assay showing normalized melt curves and difference plots for *RET*, *PAX2 *and *ALDH1A2 *genes obtained on the blinded samples**. Each sample was assayed in triplicate but the graph shows one curve for each of the 25 samples.

Twenty five samples for each gene that had been previously sequenced for the known SNPs in *RET *(rs1800860), *PAX2 *(rs11190688) and *ALDH1A2 *(rs7169289) were used for validating the HRM assay. Each sample was assayed in triplicate. The analysis of the HRM data was done independently by two observers in the following way: the curves were first auto grouped by the software based on the melt transition. The observers, who were blinded to the identity of the samples, confirmed that the Tm difference obtained between the major and minor homozygous allele fit with the Tm difference obtained in the standardization assay. The heterozygotes were identified by the shapes of their melting curves. There was no change in the classification by the observers as compared to that grouped by the software. Figure 2 shows melt profiles of the samples analyzed. The different genotypes in each of the genes were readily distinguishable in the normalized melting curves and in the difference plots (amplitude and/or shape of the curves). We observed high reproducibility of the Tm values for a given class of homozygote alleles. The Tm difference between the two homozygous alleles were 0.50°C, 0.59°C and 0.72°C for *RET*, *PAX2 *and *ALDH1A2 *genes respectively. The standard deviation for *RET*, *PAX2*, and *ALDH1A2 *were ≤ 0.06°C. All the samples for *RET *(n = 25), *PAX2 *(n = 25) and *ALDH1A2 *(n = 25) were correctly identified.

## Discussion

Sequencing is the gold standard for detecting SNPs but involves additional steps leading to a higher turn around time. High resolution melting (HRM) analysis is emerging as an alternate tool for detection of SNPs in clinical specimens [16]. HRM is a closed tube method allowing PCR amplification and analysis in a single run. The method is fast and eliminates the possibility of template contamination. Besides, a large number of samples (96 or 384 well plates) can be analyzed in a single run.

In this study, we compared the HRM technique with sequencing in order to validate the method as an alternate, rapid and robust tool to screen for known SNPs associated with small kidneys in Indian newborns. We tested two SNPs [*RET *(rs1800860) and *PAX2 *(rs11190688, G > A)] which have a detrimental effect on the kidney size while the SNP in *ALDH1A2 *gene (rs7169289) is associated with increase in kidney size. We initially optimized the assay, since small amounts of non-specific amplicons, salt concentration and template amount may contribute to variation in melt profiles.

The Tm difference among the genotypes increase as the amplicon size decreases. Hence, we used short amplicons to obtain clearly defined single peaks as shown in the derivative plots in all the three SNPs. The homozygous G/G allele for *RET*, *PAX2 *and *ALDH1A2 *melted at a higher temperature than the homozygous A/A allele due to the higher stability of the G≡C pairing compared to A = T. The resulting melting temperature difference was used to unambiguously differentiate between the two homozygous sequences.

The difference in the Tm can not be used for the determination of heterozygotes. The heterozygotes were identified based on the changes in the shapes of the melt curves as compared to the major and minor homozygous alleles. The presence of both forms of homozygotes together with heteroduplexes results in a change in the melting pattern characterized by a flatter curve shape/melting transition as seen in the normalized melting curves which is amplified on the difference plots (Figure 1). The earlier denaturation due to the presence of less stable heteroduplexes and less stable A/A homozygotes results in larger deviation from the G/G rather than to the A/A homozygote melt. All the SNPs had reproducible melting profiles.

## Conclusion

In conclusion, High Resolution Melting analysis allowed correct identification of the major and minor allele homozygotes and heterozygotes for the polymorphic variants in three genes that are associated with kidney size. HRM is a simple, rapid and cost effective technique could be used in a large population to identify babies with the risk alleles. These high risk children could then be followed up for early detection of hypertension and acquired renal dysfunction.

## Competing interests

The authors declare that they have no competing interests.

## Authors' contributions

AR designed primers, performed the assay, analyzed data and wrote the manuscript. AS prepared the blinded samples and analyzed data. TSS and KP contributed to the manuscript. AV originated the study, reviewed data analysis and co-wrote the manuscript. All authors read and approved the final manuscript.

## Pre-publication history

The pre-publication history for this paper can be accessed here:

http://www.biomedcentral.com/1471-2369/12/60/prepub
